# HETEROGENEITY IN PROGRESSION OF COGNITIVE IMPAIRMENT AND VARIATIONS IN HEALTH EXPENDITURES AND HEALTH SERVICES USE

**DOI:** 10.1093/geroni/igz038.459

**Published:** 2019-11-08

**Authors:** Wassim Tarraf, Hector M González

**Affiliations:** 1 Wayne State University, Detroit, Michigan, United States; 2 University of California, San Diego, San Diego, California, United States

## Abstract

Cognitive aging and disease (e.g. dementia) are leading public health issues as longevity increases and the US population ages. We fit generalized linear models using data from the longitudinal Health and Retirement Study (2008-2014) on (Unweighted N=1,884) participants 70-years and older who met criteria for cognitive impairment not dementia (CIND), based on Aging, Demographics, and Memory Study specification, at baseline (2008) to test how impairment reversion, stability, and transition to dementia over 8-years affect change in biennial hospitalizations, nursing-home use, and out-of-pocket expenditures (OOP). Over 8-years, 13% reverted to normal cognition, 20% remained as CIND, 21% transitioned to dementia, and 46% died. In these groups, average OOP spending at baseline was $2311 (SE=$225), $2722 (SE=$278), $2180 (SE=$228), and $3653 (SE=$322), respectively. Average OOP spending increased to $3,095, $4,720, and $11,548 by the 8th year for those that reverted, stayed stable, and transitioned, respectively. Average OOP spending at the wave preceding death was $11,600. We observed substantial increases in nursing home use that were particularly pronounced among those that transitioned to dementia (Baseline Probability=0.04 increasing to 0.37 over 8-years) or died (0.09 increasing to 0.35 over 6-years), and similar but less pronounced differences in patterns of inpatient hospitalizations. Estimates were only slightly modified through adjustments to sociodemographic characteristics and comorbid conditions. We discuss how healthcare policy and clinical interventions focusing on early identification of impairment can potentially lead to improved and more efficient healthcare if better understanding of heterogeneities in impairment and cognitive disease progression is achieved.

